# A Noninvasive Diagnostic Approach for Identifying Spontaneous Coronary Artery Dissection (SCAD) in Young Women: A Case Report and Review of the Literature

**DOI:** 10.7759/cureus.79167

**Published:** 2025-02-17

**Authors:** Michael S Nasr, Marc Haber, Samer R Nasr

**Affiliations:** 1 Department of Medicine, American University of Beirut, Beirut, LBN; 2 Department of Cardiology, Mount Lebanon Hospital, Hazmiyeh, LBN

**Keywords:** case study, diagnosis, epidemiology, noninvasive cardiology, short reviews, spontaneous coronary artery dissection, treatment

## Abstract

A 19-year-old female patient presented with an acute onset of substernal chest pain, accompanied by dyspnea and diaphoresis while walking to the gym. She was able to start her routine, but the pain worsened. She was taken to the emergency room, where an electrocardiogram was performed, and no irregularities were observed, while the troponin test was significantly elevated. Imaging studies, including a Doppler echocardiogram and an emergent CT of the chest to rule out aortic dissection, returned normal results. The pain subsided after two hours, but troponin kept increasing; consequently, spontaneous coronary artery dissection (SCAD) was suspected. In view of the young age of the patient, it was decided to refrain from performing a coronary angiogram, intravascular ultrasound, or optical coherence tomography. A CT coronary angiography scan did not show any anomaly. A cardiac magnetic resonance imaging showed a clear subendocardial enhancement indicative of a myocardial infarction. A diagnosis of SCAD was confirmed, and no further testing was done. The patient was started on aspirin and beta-blockers, and advised to perform only minimal to moderate exercise and to consult a healthcare physician immediately if the episode recurs. CT angiogram ruled out fibrodysplasia of the renal arteries, and plasma metanephrines were normal. This case shows that with the advancement of noninvasive techniques, there is probably no need for invasive measures to diagnose SCAD in stable patients.

## Introduction

Epidemiology of SCAD

Spontaneous coronary artery dissection (SCAD) is defined as a dissection of the epicardial coronary artery that occurs without association with atherosclerosis, trauma, or iatrogenic causes. In cases of SCAD, myocardial injury is primarily due to coronary artery blockage, which is caused by the formation of an intramural hematoma or disruption of the intima. This mechanism differs from the typical atherosclerotic plaque rupture or intraluminal thrombus seen in other forms of coronary artery disease [1].

SCAD was historically viewed as a rare and frequently fatal cause of acute coronary syndrome (ACS), myocardial infarction (MI), and sudden cardiac death, particularly in women during the peripartum period [2-4]. However, recent advancements, such as improvements in intravascular imaging and the introduction of SCAD-specific angiographic classifications, combined with increased awareness among healthcare providers and patients through social media, have revealed that SCAD is more prevalent than once believed, especially among younger women [1].

The prevalence of SCAD is unclear, as it is frequently misdiagnosed or underdiagnosed [1]. However, recent studies have reported the distribution of SCAD in several regions. A study conducted in Germany reported that SCAD was identified in 1.1% of patients diagnosed with coronary artery disease. Among those presenting with acute MI, the incidence rose to 2.9%, and it reached 4.2% in patients with unstable or postinfarction angina [5]. More recent studies suggest that SCAD incidence ranges between 0.12% and 4% in patients who present with ACS or undergo coronary angiography [6]. In the United States, a study reported a significant increase in SCAD incidence, rising from 4.95 per million discharges in 2010 to 14.8 per million in 2017 [7].

Missed diagnoses of ACS in younger women are often due to low clinical suspicion, even when classic symptoms are present, along with the technical limitations of coronary angiography and the limited awareness of SCAD among healthcare professionals [1]. SCAD is most frequently observed in patients with minimal or no traditional cardiovascular risk factors [8-10]. While women diagnosed with SCAD are typically in the fourth and fifth decades of life, cases in younger populations have also been reported [1].

Additionally, although SCAD has been documented in various racial and ethnic groups, most reported cases involve patients from the White ethnicity [1]. SCAD can be precipitated by various triggers, such as hormonal fluctuations, emotional stress, or physical strain like excessive coughing or vomiting, which increase the shear stress on the arterial walls. Combined with preexisting vascular conditions, these triggers may lead to arterial disruption. Several diseases, such as fibromuscular dysplasia and systemic inflammatory conditions, are thought to contribute to vascular damage, though they do not provide a definitive cause [11]. It is important to note that patients with SCAD present distinct combinations of risk factors compared with patients presenting with cardiovascular diseases [12].

Furthermore, a systematic review reported that the overall SCAD mortality rate was estimated at 1% over an average follow-up of 33 months. Male sex was associated with a 3.5 times increased risk of mortality, while current and past smoking was linked to a 15 times higher mortality risk [13].

Pathophysiology of SCAD

SCAD is classified as an idiopathic condition and is recognized as an important cause of ACS, particularly in young, healthy women with minimal to no cardiovascular risk factors [14]. It is also frequently associated with pregnancy, further distinguishing it from other forms of coronary artery disease [15]. The mechanism of coronary flow obstruction in SCAD varies, involving either an intimal flap, which is typically identifiable on coronary angiography, or the formation of an intramural hematoma that compresses the vessel lumen externally. However, intramural hematomas can sometimes remain undetected in coronary angiography, particularly when they affect distal segments, necessitating the use of cardiac computed tomography angiography (CTA) or other advanced imaging techniques for diagnosis [14]. In cases where the hematoma is located distally, it may still be missed, making coronary arteriography with intracoronary imaging, such as intravascular ultrasound (IVUS) or optical coherence tomography (OCT), necessary. These procedures, however, carry the inherent risk of exacerbating the dissection due to wire manipulation or contrast injection [16].

The coronary artery distribution of SCAD is well documented. The left anterior descending (LAD) artery is the most commonly affected vessel, involved in 32%-46% of cases. In terms of specific coronary territories, SCAD affects the LAD along with its diagonal and septal branches in 45%-61% of cases. The circumflex artery, along with the ramus and obtuse marginal branches, is involved in 15%-45% of cases. The right coronary artery, including the acute marginal, posterior descending, and posterolateral branches, is affected in 10%-39% of cases. In contrast, the left main artery is less commonly involved, seen in up to 4% of cases. Notably, SCAD predominantly affects the mid to distal segments of the coronary arteries, with the proximal segments involved in less than 10% of cases. Around 9%-23% of cases present with multivessel SCAD [1].

A multimodality diagnostic approach can be considered in patients who do not present with ST-segment elevation on electrocardiogram (ECG), where pain has resolved, and where the physician assesses that the vessel is not occluded. This approach includes the use of clinical data, biomarkers, and noninvasive imaging techniques such as echocardiography, cardiac CTA, and cardiac magnetic resonance (CMR) [17].

Diagnostic modalities

The diagnosis of SCAD relies on a range of imaging techniques, which are broadly categorized into invasive and noninvasive approaches. Invasive methods, such as coronary angiography, are the gold standard for diagnosis but cannot display arterial wall structures. Intracoronary imaging methods, such as OCT and IVUS, supplement angiography by visualizing arterial wall layers, improving SCAD [18].

However, these techniques carry risks, including vessel injury and exacerbation of dissection, making them less favorable in certain patient populations. Noninvasive modalities, including cardiac CTA, CMR, and echocardiography, offer safer alternatives for diagnosing SCAD. These methods could evaluate myocardial injury and rule out differential diagnoses while avoiding the complications associated with invasive procedures [18].

Treatment and management

Stable patients with SCAD are generally managed conservatively, as many dissected segments heal on their own. Treatment guidelines are largely informed by expert opinion in the absence of randomized trials. Management typically mirrors that of ACS, including the use of dual antiplatelet therapy, heparin, and beta-blockers to preserve lumen patency. Though concerns exist about delayed healing and dissection extension, glycoprotein IIb/IIIa inhibitors may be utilized. Thrombolytic agents, however, are contraindicated due to increased bleeding risk [19].

Ethical considerations

Before preparing this case report, informed consent was obtained from the patient. All personal information was deidentified to ensure confidentiality and protect the patient’s anonymity. Every precaution was taken to safeguard the patient’s privacy, and all details included in the report were presented in a manner that complies with ethical guidelines for medical case reports.

## Case presentation

**Figure 1:**
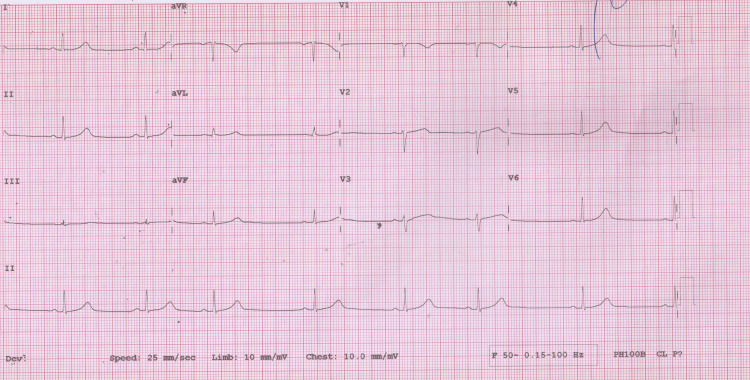
Electrocardiogram demonstrating the absence of abnormalities

**Figure 2:**
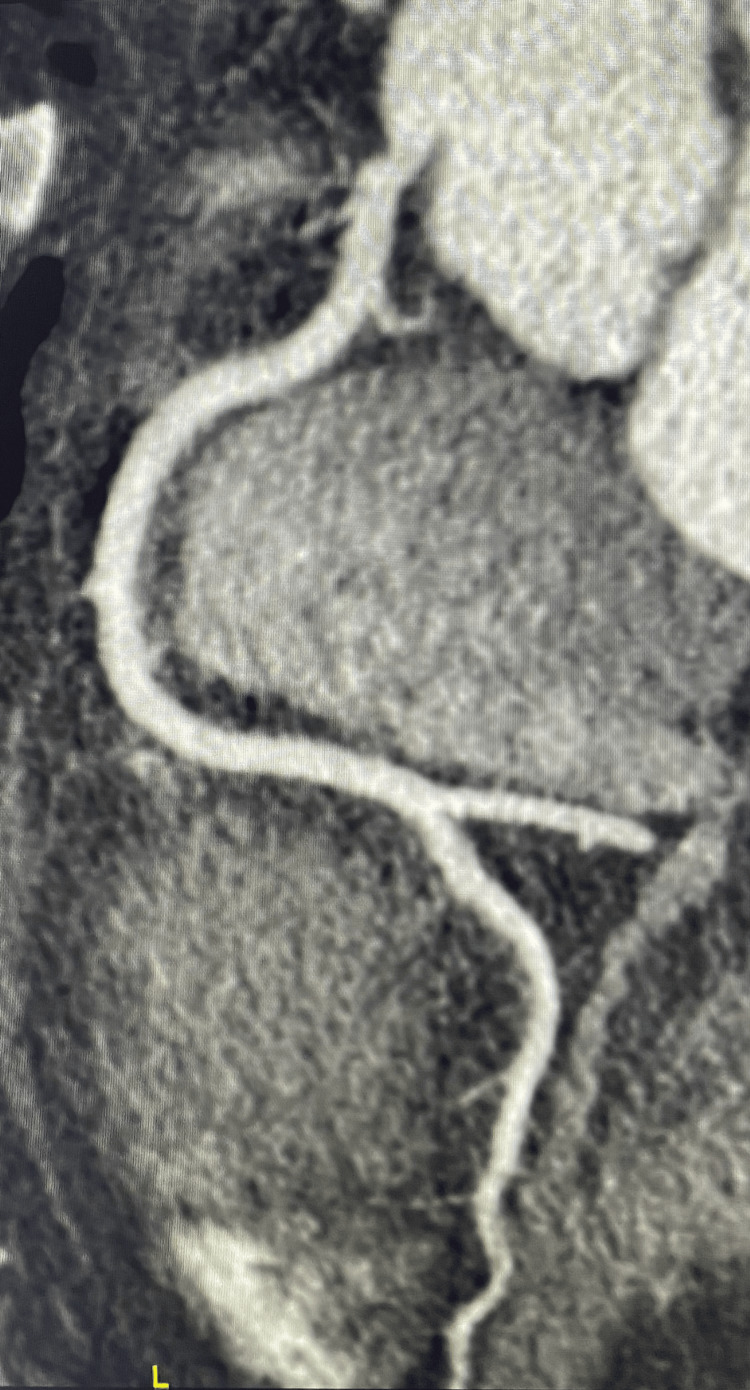
CT coronary angiogram showing normal right coronary artery CT: computed tomography

A 19-year-old previously healthy female patient presented to the emergency room with substernal chest pain. The pain was severe, with a self-reported intensity of 10 on a scale of increasing severity from 1 to 10. The pain irradiated to the back between the shoulder blades and was associated with dyspnea and diaphoresis. Severe pain started two hours before the presentation to the emergency room as she was walking to the gymnasium on an uphill path, but she disregarded her symptoms, hoping that they might dissipate on their own. The patient reported consistent weightlifting for two years. Upon arrival, she sat down, the intensity of the pain diminished, and she started her regular exercise routine consisting of upper body weightlifting. During training, the pain recurred, and she felt a tearing sensation in her chest, prompting her to visit the emergency room in a private car. On arrival, the patient appeared distressed but was fully alert and oriented. She weighed 75 kg and was 184 cm tall. The blood pressure was 130/80 mmHg, the heart rate was 75 bpm, and she was afebrile. A complete physical examination and a 12-lead ECG showed normal results (Figure 1). An echocardiogram Doppler in the emergency room showed no valvular abnormalities, normal aorta, pulmonary pressure, and right and left ventricles with no signs of pericardial effusion. A portable chest X-ray was normal. Aortic dissection was suspected, and a CT angiogram was performed, which returned normal results (Figure 2). The blood tests returned significant for troponin I at 30 times the upper limit, and C-reactive protein (CRP) was normal, while the rest of the laboratory workup was normal. Treatment was initiated in the emergency room. She received intravenous paracetamol and was prescribed bisoprolol (5 mg) daily. A nitroglycerin patch was placed on the chest. A repeat ECG remained normal. The pain subsided progressively, and she was relieved of her chest pain one hour after the presentation, after which a second troponin I test showed even higher levels than the first. Accordingly, she was transferred to the coronary care unit with a differential diagnosis of myocarditis vs. spontaneous SCAD.

**Figure 3:**
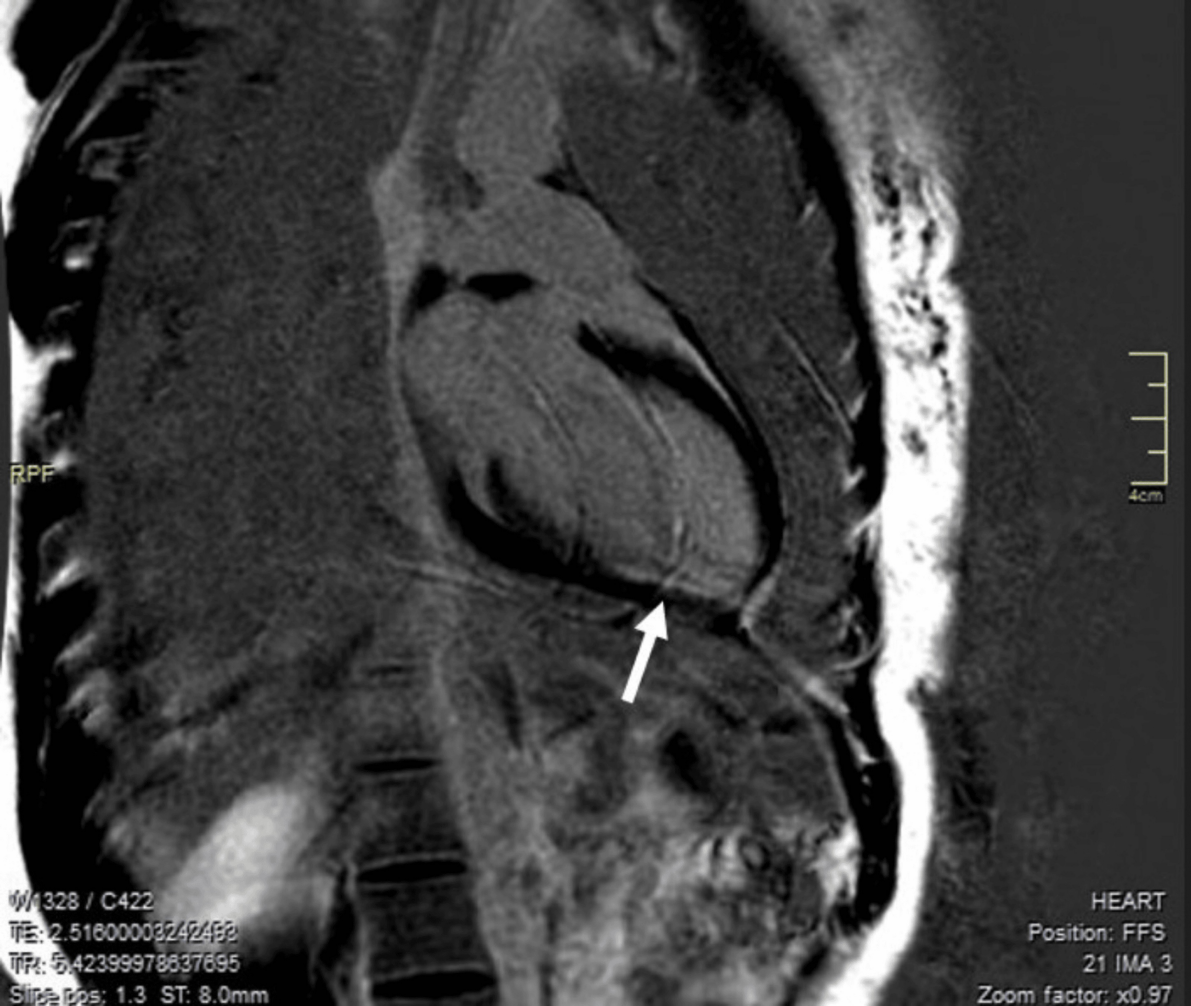
Cardiac MRI showing scaring suggestive of a small myocardial infarction (white arrow) MRI: magnetic resonance imaging

She remained asymptomatic the next day, and a repeat CRP test returned negative results, alongside elevated troponin I levels. The highest troponin I value recorded the following day was 1.72 ng/mL, which exceeded the laboratory's upper reference limit for high-sensitivity troponin I of 0.03 ng/mL. A complete echocardiogram and cardiac CT scan did not show any anomalies the next day. A cardiac magnetic resonance imaging (MRI) showed subendocardial enhancement typical of an MI in the inferior wall with no evidence of myocarditis (Figure 3).

The diagnosis of SCAD was confirmed, and a coronary angiogram was deemed unnecessary. Muscular fibrodysplasia and pheochromocytoma were excluded through the Doppler ultrasound of the renal arteries and measurement of plasma metanephrines. The patient was consequently discharged on bisoprolol 5 mg daily and aspirin and was asked to stop weightlifting but continue low-to-moderate exercise. She was instructed to consult with a healthcare physician if pain recurs. She followed up monthly in the following three months and remains entirely asymptomatic to date. Since SCAD may recur, especially in the case of pregnancy, the patient was instructed to remain consistent with her follow-up visits.

## Discussion

This case highlights the critical role of noninvasive diagnostic modalities in identifying SCAD in a young, stable patient without high-risk features. SCAD, often underdiagnosed or misdiagnosed, poses unique challenges due to its variable presentation and the potential risks associated with invasive diagnostic procedures. By employing noninvasive imaging, this case underscores a safe and effective alternative to traditional invasive approaches, with implications for the broader management of SCAD, particularly in low-risk populations. The decision to pursue a noninvasive diagnostic approach was further supported by the patient's clinical stability, as she was chest pain-free and became totally asymptomatic.

Diagnosing SCAD involves selecting appropriate tools that balance accurate detection with minimizing procedural risks. Due to similarities in clinical presentation with other ACS, imaging is essential for confirmation, utilizing methods that differ in their sensitivity, specificity, and safety.

ECG and SCAD

Patients with SCAD will often have ECG changes upon presentation indicative of ischemia. Specifically, 30%-40% of patients present with ST-elevation myocardial infarction (STEMI), and up to 70% present with non-STEMI [20,21]. Several case series have reported that ECGs can show STEMI in up to 36% of SCAD cases. In patients with non-STEMI, the most frequent ECG changes include T wave inversion, occurring in up to 24% of cases, followed by ST depression in 6% of cases. Normal ECG was less typical but is found in rare cases [21]. Additionally, 8.3% of patients can present to the hospital with arrhythmias, such as ventricular tachycardia or fibrillation. These findings conclude that SCAD mimics atherosclerotic ACS, necessitating further investigations to confirm the diagnosis [20,21].

Echocardiography and SCAD

Echocardiography in patients presenting with SCAD has a limited role in guiding or confirming the diagnosis. Although echocardiography may show reduced strain or regional wall motion abnormalities in the area related to the affected coronary artery region, these findings are not definitive for an SCAD diagnosis [22]. Some case series showed that 67% of patients with SCAD presented with regional wall abnormalities with a preserved ejection fraction, while 44%-49% of patients presented with a reduced left ventricular ejection fraction of less than 50% [18,22]. Furthermore, a normal echocardiography result does not help differentiate SCAD from other conditions, as these findings are nonspecific to nonatherosclerotic. Thus, while echocardiography alone is insufficient for a definitive diagnosis, it can sometimes assist in ruling out or ruling in another differential diagnosis [21,23].

Cardiac biomarkers and SCAD

Patients with SCAD typically present to the emergency room with symptoms identical to those of atherosclerotic ACS, making the clinical distinction between the two entities challenging [20,23,24]. Patients with SCAD experience typical chest pain and will have elevated cardiac biomarkers in 72%-97% of cases, with only 0.5%-4% of cases not exhibiting elevated biomarkers [20,21,24]. This elevation is mostly explained by the physiopathology of SCAD, which involves the formation of an intramural hematoma that can expand and obstruct the true lumen, thereby causing these symptoms [21,24]. It is, therefore, difficult to distinguish between SCAD and atherosclerotic ACS clinically, with the only difference being the phenotype. Unlike patients with ACS, who typically have atherosclerotic risk factors, those with SCAD often present at a younger age, have few to no traditional cardiovascular risk factors, and have precipitating factors such as exercise or emotional stress [21]. Consequently, it is important to conduct a full and thorough medical history and proceed with further investigative modalities, such as imaging, to help differentiate the two conditions and confirm the diagnosis of SCAD [21,23].

Coronary angiogram, percutaneous coronary intervention, IVUS, OCT, and SCAD

Percutaneous coronary intervention is recommended only for patients who exhibit SCAD symptoms and signs of ongoing myocardial ischemia, a large area of myocardium in jeopardy, and reduced antegrade flow [1]. Since many patients with SCAD are free from chest pain and do not show signs of ongoing ischemia, the need for a coronary angiogram as a tool for diagnosis might be questioned. A coronary angiogram is usually followed by IVUS or OCT, which necessitates threading a wire in the coronary artery [25]. This invasive procedure entails a number of risks and complications that include blood vessel injury, excessive bleeding, heart attack, infection, irregular heart rhythms, kidney damage from the dye used, allergic reactions to the dye or medications, and stroke [26]. The European Society of Cardiology 2023 guidelines do not recommend IVUS or OCT unless a coronary intervention is to be undertaken [27]. It is postulated that clinical presentation, ECG, biological markers, an echocardiogram Doppler with cardiac CT scan, and cardiac MRI are enough to establish the diagnosis of SCAD, and there is no need to perform a coronary angiogram in young populations.

Cardiac computed tomography and SCAD

In patients with stable coronary artery disease, when using intracoronary angiography and fractional flow reserve as a reference, coronary CTA can identify anatomical CAD at a pretest probability greater than 58% and rule out CAD at a pretest probability less than 80%. Within these pretest probability thresholds, the sensitivity of coronary CT angiography is 97%, with its specificity reaching 78% [28].

With SCAD, multiple case series reported inconsistent results. The dissection usually involves the small distal coronary arteries, which may be beyond the spatial resolution of coronary CT angiography, especially if the vessel is less than 1.5 mm. Additionally, various findings on coronary CT angiography in patients with SCAD have been identified across multiple case series. Notably, the absence of atherosclerotic plaque by itself makes a diagnosis of SCAD more likely [29]. Diagnostic indicators of SCAD include abrupt luminal stenosis, tapered luminal stenosis, luminal occlusion and intramural hematoma, and dissection flaps. However, the absence of these indicators does not rule out the disease [29,30]. Furthermore, when coronary CT angiography results are normal, SCAD cannot be ruled out. In such cases, complimentary CMR is recommended if SCAD is suspected [29].

CMR and SCAD

Over the past years, CMR has become a valuable resource for diagnosing MI, helping evaluate the heart function and structure, measuring the extent of the infarct, and assessing the risk for subsequent cardiovascular events. CMR is particularly useful in cases where the cause of the myocardial damage is still unknown, as it can accurately diagnose ACS by outlining the features of the myocardial tissue and providing imaging evidence of acute MI. Although the role of CMR in SCAD remains unclear, a few case series have shown that it might improve the understanding of this understudied condition, helping in its diagnosis and in predicting its outcomes [31,32].

The CMR findings that are indicative of ischemia are T1-weighted late gadolinium enhancement (LGE), myocardial edema (hyperintense on T2-weighted images), microvascular obstruction (MVO; hypointense core within the hyperintense LGE infarct zone), and perfusion defect suggesting intramyocardial hemorrhage (hypointense area in the center of the hyperintense zone in edema sequences) [32,33].

A few case series reviewing CMR findings in SCAD patients reported that most cases had ischemia-related changes on the CMR, predominantly LGE with varying degrees of severity (transmural, subendocardial, or patchy). The location of LGE was associated with the localization of the artery affected by SCAD. Larger infarcts were correlated with MVO and intramyocardial hemorrhage. However, a minority of cases in these case series showed no abnormalities in the CMR. Therefore, a normal CMR does not rule out SCAD [32,34]. Moreover, no CMR characteristics were found to predict high-risk features in patients with recurrent SCAD [34]. Consequently, CMR can help differentiate SCAD from other entities that can mimic MI, such as Takotsubo syndrome or myocarditis. Additionally, with adjunctive imaging modality, like cardiac CT angiography, which helps visualize the coronary arteries, CMR can be a beneficial tool to diagnose SCAD without using more invasive methods that carry a greater risk of complications.

## Conclusions

SCAD frequently occurs in young asymptomatic women. It can sometimes be fatal, especially when it occurs or extends to the proximal part of the coronary arteries, such as the proximal left anterior descendant or even left-main. In such situations, clinical and electrical signs of continuous ischemia are evident, and coronary interventions are warranted. On the other hand, when SCAD occurs more distally and is not accompanied by clinical or electrical signs of ischemia, we believe that a coronary angiogram is not warranted since it may lead to unnecessary interventions. This may worsen the long-term prognosis of this young population by altering the natural progression of the disease. In these situations, a noninvasive approach combining a coronary CT scan and a cardiac MRI should be enough to establish the diagnosis and guide medical therapy.

To conclude, this case exemplifies a growing trend toward prioritizing patient safety through noninvasive diagnostic techniques, particularly in low-risk populations. These techniques may reduce complications and healthcare costs associated with invasive procedures.
